# Editorial: Advancing cancer therapy: innovative strategies targeting immune evasion and metabolic modulation

**DOI:** 10.3389/fmed.2025.1677300

**Published:** 2025-08-29

**Authors:** Mónica Teotónio Fernandes, Ana Luísa De Sousa-Coelho, Andrés Méndez-Lucas

**Affiliations:** ^1^Escola Superior de Saúde, Universidade do Algarve (ESSUAlg), Faro, Portugal; ^2^Algarve Biomedical Center Research Institute (ABC-Ri), Universidade do Algarve (UAlg), Faro, Portugal; ^3^Physiological Sciences Department, School of Medicine, University of Barcelona (UB), Barcelona, Spain; ^4^Bellvitge Biomedical Research Institute (IDIBELL), L'Hospitalet de Llobregat, Spain

**Keywords:** cancer therapy, biomarkers, immunotherapy, metabolism, therapy resistance, personalized medicine

Cancer remains one of the leading causes of death worldwide, with both incidence and mortality continuing to rise despite advances in diagnosis and treatment (1). While early-stage cancers often respond to conventional therapies, advanced and recurrent tumors frequently develop resistance, limiting long-term therapeutic efficacy (2).

Two fundamental hallmarks of cancer, immune evasion and metabolic reprogramming, enable tumors to thrive in hostile microenvironments (3, 4). Although immunotherapies have revolutionized cancer care, a significant proportion of patients either fail to respond or acquire resistance over time (5). In parallel, altered tumor metabolism is increasingly recognized as a promising therapeutic target, particularly for enhancing responses to immunotherapy (7).

This Research Topic highlights recent advances that move beyond traditional treatment. Collectively, the nine featured articles provide valuable insights into the interplay between immunity and metabolism in cancer, exploring strategies to overcome therapeutic resistance and improve clinical outcomes across diverse cancer types.

Several contributions in this Research Topic showcase innovative strategies in immuno-oncology, with a particular focus on integrating biomarkers, imaging techniques, and immune modulation to propel the development of personalized cancer therapies.

Wei et al. addressed a key clinical question in immuno-oncology: does the timing of immune checkpoint inhibitor (ICI) therapy influence outcomes in advanced esophageal squamous cell carcinoma? Their study revealed that, while early immunotherapy does not significantly improve overall survival, it does prolong progression-free survival, particularly in defined patient subgroups. These findings underscore the need to personalize not only the type of treatment but also its timing, especially in settings where biomarkers like PD-L1 are not routinely available (Wei et al.).

In the pursuit of biomarkers to predict immunotherapy response, a cornerstone of precision oncology, Wang Y. et al. explored the prognostic significance of CD74 expression in non-small cell lung cancer (NSCLC) and developed a radiomics-based machine learning model to predict CD74 levels from contrast-enhanced CT images. Their results demonstrate that high CD74 expression correlates with improved overall survival and enhanced antitumor immune activity. The radiomics models achieved strong predictive performance, offering a non-invasive method to stratify patients. This work positions CD74, a membrane glycoprotein involved in immune signaling, as both a prognostic biomarker and a potential therapeutic target in NSCLC, while showcasing the promise of AI-driven imaging biomarkers in precision oncology (Wang Y. et al.). Extending this theme, Xie et al. introduced another radiomics-based machine learning approach to predict response to neoadjuvant immunochemotherapy in advanced NSCLC. By analyzing pre-treatment CT scans, they developed a radiomic signature capable of distinguishing responders from non-responders. Together, these studies highlight radiomics as a non-invasive, scalable tool to guide patient selection and optimize immunotherapy outcomes (Xie et al.).

Focusing on immune evasion in breast cancer, Ding et al. examined how the natural compound resveratrol sensitizes breast cancer cells to natural killer (NK) cell-mediated cytotoxicity. Their work revealed that resveratrol downregulates miR-17-5p, leading to MINK1/JNK/c-Jun pathway activation and upregulation of the NKG2D ligand ULBP2. This enhanced NK cell recognition and killing of tumor cells both *in vitro* and *in vivo*, suggesting that dietary or pharmacologic interventions could potentiate innate immune clearance mechanisms (Ding et al.).

Finally, Wang B. et al. provide an insightful review of the cyclic GMP-AMP synthase-stimulator of interferon genes (cGAS-STING) pathway in the anti-tumor innate immune response and the use of STING agonists to overcome resistance to conventional therapies. The authors report mechanisms by which STING agonists have the potential to convert “cold” tumors, which lack immune cell infiltration, into “hot” tumors that are more responsive to immunotherapy, present a broad range of STING agonists categories, and discuss several challenges that must be addressed to fully realize the clinical potential of this approach (Wang B. et al.).

Among the selected contributions, other articles delve into cancer metabolism and emerging technologies that are shaping the future of personalized oncology.

The glycocalyx is a glycan-rich layer on the cell surface, with a distinct composition in tumor cells compared to healthy ones. On T cells, glycans regulate key functions and interact with glycan-binding proteins involved in tumor progression. Many immune receptors, such as PD-1, are glycosylated, affecting their stability, ligand binding, and recognition by therapeutic antibodies (6). These topics are discussed in Schuurmans et al., who reviewed the interplay between tumor glucose metabolism and T cell glycocalyx, which is essential for adequate T cell activation and may represent a relevant target to improve anti-tumor T cell biology.

The role of metabolic reprogramming in cancer progression and resistance to therapy in NSCLC was explored in a comprehensive review by Cai et al. After identifying NSCLC key metabolic vulnerabilities, the authors discuss how these can be exploited with drugs and/or compounds that target the glucose, mitochondrial, lipid, and amino acid metabolism pathways, which may be combined with immunotherapies (Cai et al.). The authors also highlight the use of single-cell and spatial metabolomics to identify metabolic subtypes, which could lead to more personalized treatments.

These emerging technologies were applied in an integrative original article, which analyzed single-cell sequencing and spatial transcriptomics data from hepatocellular carcinoma (HCC) sourced from databases (Xi et al.). Xi et al. used computational tools to map the expression of glucose metabolism-related genes and explored the spatial dynamics of glucose metabolism in HCC. From *in vitro* assays, G6PD, the rate-limiting enzyme of the pentose phosphate pathway, was identified to be involved in HCC progression, associated with glutathione metabolism and ROS production (Xi et al.).

Finally, a review by Fan et al. explores in depth the molecular subtyping of pancreatic cancer, integrating multiple layers of data encompassing gene mutations, genomics, transcriptomics, proteomics, metabolomics, and immunomics. They concluded that the integration of multi-omics approaches is critical for developing personalized treatment approaches and improving the clinical outcomes (Fan et al.).

This Research Topic showcases innovative research that collectively advances our understanding of how cancers escape immune detection and rewire metabolism to sustain growth. The nine featured studies offer mechanistic insights and propose translational strategies ranging from STING pathway activation to targeting metabolic vulnerabilities. We thank all the authors and reviewers for their valuable contributions and hope this Research Topic inspires continued efforts to bridge immunology, metabolism, and oncology for more effective and durable cancer therapies.
